# Functional/dissociative seizures as a neuroenergetic deficit syndrome: a brain economy failure hypothesis

**DOI:** 10.3389/fneur.2026.1865708

**Published:** 2026-07-03

**Authors:** Lana Higson

**Affiliations:** 1 Department of Neuroscience, School of Translational Medicine, Monash University, Melbourne, VIC, Australia; 2 Department of Neurology, Alfred Hospital, Melbourne, VIC, Australia

**Keywords:** cerebral hypoxia, default mode network, functional neurological disorder, functional/dissociative seizures, ictal haemodynamics, neuroenergetics, psychogenic non-epileptic seizures, sex differences

## Abstract

Functional/dissociative seizures are debilitating and account for up to one-third of presentations to epilepsy monitoring units. Despite this, their pathophysiological mechanism remains poorly understood and available treatments lead to seizure freedom in fewer than half of patients. It is proposed here that functional/dissociative seizures may involve a neuroenergetic deficit syndrome or a brain economy failure where an episodic, insufficient supply of cerebral oxygen is met with an inability of the brain to proactively reorganise the network response. When cerebral oxygen delivery falls below a critical threshold and the brain cannot mount an adaptive reorganisation, neural networks fall sequentially in order of energetic cost, which produces the characteristic symptom profile of functional/dissociative seizures. The subsequent response - convulsions, stiffening, postural collapse - is a physiological rescue mechanism to ensure restoration of cerebral perfusion through skeletal muscle pump activation. This hypothesis accounts for several features of functional/dissociative seizures that are not adequately explained by existing biopsychosocial models. These include the overlap of symptoms with controlled hypoxia; the predominance in females (for the first time linked to documented sex differences in physiological responses to hypoxia); the shift in female-to-male ratio at puberty; the relatively favourable prognosis in paediatric populations; the relapsing–remitting course of the condition; and the heterogeneity of clinical presentations predicted by hub-first, cost-ordered network failure. The paper describes the evidence supporting the hypothesis, its discriminating predictions, and the experimental and clinical approaches for testing it.

## 1 Introduction

It is important to clarify that the neuroenergetic hypothesis is about *cerebral oxygen insufficiency* (i.e., a mismatch between local cerebrovascular oxygen delivery and metabolic demand at the network level) rather than systemic hypoxia. Normal peripheral oxygen saturation does not exclude cerebral oxygen insufficiency, which can arise from impaired cerebrovascular autoregulation, reduced cerebrovascular CO_2_ reactivity, or local metabolic demand exceeding delivery capacity in the absence of systemic desaturation.

Functional/dissociative seizures (FDS) are a highly disabling condition that disproportionately affects females with an overall prevalence of ~0.1% of the population (1). Antiseizure medications have no effect on debilitating symptoms characterised by sudden changes in awareness, movement, and/or behaviour that include sudden loss of consciousness, convulsions, and collapses (2). Despite decades of research, there is no gold standard treatment, primarily because there is little understanding of their precise mechanism (3). Functional/dissociative seizures (FDS) sit across several diagnostic frameworks. The ICD-11 places them under dissociative neurological symptom disorder with non-epileptic seizures; the DSM-5 places them under functional neurological symptom disorder (conversion disorder) with attacks or seizures. A recent ILAE proposal has recommended functional/dissociative seizures as the preferred label - a reflection of the way the condition has come to sit between neurological and psychiatric framings rather than being claimed by either (3). With a mortality rate over twice that of the general population (4) and significant health care resource use due to diagnostic delays (on average ~7 years), misdiagnosis, and persistent symptoms (5), there is a critical unmet need to uncover why and how FDS occur. Because they occur without any electrical storm in the brain, psychological factors like stress and anxiety are thought to play a role, however, substantial knowledge gaps limit the development of effective treatment, including: (i) how do psychological factors manifest as physical seizure symptoms? and (ii) why do overt signs of stress not routinely precede functional seizures? (6).

Considerable heterogeneity within the disorder has proved challenging for establishing a universal aetiological model of FDS (3). Initially conceptualised as ‘hysteria’ in the traditional Freudian sense, the dominant explanatory frameworks of FDS have predominantly been psychological in nature and include the activation of dissociated materials (7), hard-wired responses such as “panic without panic” (8), physical manifestations of emotional distress (9), or learned behaviours (10). Brown and Reuber’s Integrative Cognitive Model (ICM) proposes that learned seizure representations are activated under conditions of arousal and attentional dysregulation (10); while predictive coding models frame FDS as failures of precision-weighted prediction error resolution under conditions of psychological threat (11). While these accounts have generated important clinical insights, they fail to resolve several challenges, including: (i) the approximately threefold female preponderance, which has been accommodated retrospectively rather than predicted from biological first principles (12); (ii) the selectivity of FDS (most people with recognised risk factors never develop FDS); (iii) the full FDS symptom profile is reproducible in healthy people under conditions of controlled cerebral hypoxia (13); (iv) peri-ictal behavioural analyses demonstrate that overt psychological stressors do not routinely precede FDS onset (6); (v) existing accounts do not specify the biological pathway through which psychological triggers produce physiological FDS symptoms, leaving the conversion mechanism unexplained at the mechanistic level; and (vi) psychological treatments have demonstrated limited and inconsistent reductions in seizure frequency (14).

The hypothesis is advanced as a candidate mechanism in FDS pathogenesis, not as a complete account of the disorder. Given the clinical and aetiological heterogeneity of FDS, it is unlikely that any single mechanism accounts for all presentations. What is proposed here is that episodic insufficiency of cerebral oxygen delivery, combined with impaired adaptive neural network reorganisation, contributes to the generation of FDS episodes - potentially in a subgroup of patients, or as one operative pathway among several. The account is positioned as complementary to existing cognitive and psychological models of FDS (including the ICM), rather than as a replacement for them. From this perspective, the neuroenergetic mechanism may constitute part of the biological substrate through which cognitive and psychological vulnerabilities translate into episodes. Seven testable predictions are listed in Section 2.8. Failure of the haemodynamic predictions would constrain the hypothesis but leave the surrounding biopsychosocial account untouched.

## 2 The hypothesis

Functional/dissociative seizures (FDS) are proposed to represent a neuroenergetic deficit syndrome (NEDS) - a brain economy failure. The brain is the body’s most metabolically demanding organ, and its energy consumption is unevenly distributed across regions and networks, meaning that some networks are far more metabolically expensive to sustain than others (15). FDS episodes, their phenomenology, their resolution, and their recurrence are hypothesised to share a common mechanism - insufficient cerebral oxygen delivery coinciding with a failure of the brain’s adaptive response to that insufficiency. This mechanism is proposed to unfold in five stages, outlined below. In what follows, declarative descriptions of the stages should be read as the hypothesis’s proposed mechanism rather than as established findings; the supporting evidence and its limitations are discussed in Sections 2.7 and 3.

### 2.1 Stage I: background vulnerability and eroded oxygen reserve

Some people may have a chronically reduced cerebral oxygen reserve, which is not sufficient alone to cause symptoms, but enough to lower the threshold at which FDS occur. This underlying vulnerability may stem from multiple interacting pathways, including but not limited to impaired oxygen delivery caused by cardiovascular inefficiency, reduced oxygen-carrying capacity due to anaemia, recurrent nocturnal desaturation and intermittent hypoxic brain exposure from obstructive sleep apnoea (OSA), a reduction in expiratory reserve volume and resting oxygenation due to obesity, chronic mild hypocapnia and associated cerebrovascular vasoconstriction caused by dysfunctional breathing patterns, and/or a raised resting metabolic demand due to heightened sympathetic nervous system activation (16–21). Supporting this, moderate to severe sleep-disordered breathing has been identified in approximately 29% of FDS patients admitted for video-EEG monitoring, comparable to the rate observed in epilepsy patients in the same setting (22). This finding suggests that nocturnal hypoxic burden is substantially prevalent in patients presenting for seizure monitoring, although whether this rate exceeds that of the general population remains unknown. In people with recurrent FDS episodes, repeated oxygen desaturation may create a new vulnerability via the production of reactive oxygen species (ROS) during each hypoxic episode, the generation of which increases in proportion to oxygen saturation (SpO_2_) depression and is not fully cleared before the next episode. At the same time, obesity may worsen the desaturation itself, and the sympathetic hyperactivation of chronic stress can raise the metabolic demand against which that desaturation is felt. The interaction is thought to be cumulative in a way that the individual factors, considered separately, would not predict (23, 24). Consequently, psychological burden may load physiological vulnerability directly rather than through a separate psychological route.

Chronic stress and early adversity exert effects on cerebral metabolism that are relevant to the vulnerability account independently of their psychological consequences. Early neglect and maltreatment have documented effects on hypothalamic–pituitary–adrenal (HPA) axis regulation, sympathetic tone, and neuroinflammatory burden that persist into adulthood and alter the metabolic environment in which network reorganisation must occur (23, 25). These biological consequences of adverse early experience sit within the biopsychosocial framework rather than outside it - they represent the biological mechanism through which psychological and social adversity narrows the neuroenergetic margin.

One question the hypothesis raises is the effect of chronic psychotropic medication use on the threshold. Selective serotonin reuptake inhibitors (SSRIs) have cerebrovascular effects, with serotonin acting on receptor subtypes that regulate vessel diameter and cerebral blood flow (26). Benzodiazepines bind GABA-A receptors expressed on brainstem respiratory neurons; their characteristic effect is a reduction in tidal volume and a flattening of the ventilatory CO_2_ response slope, with associated effects on resting breathing pattern (27). These physiological effects are independent of the conditions the drugs treat. In a population where medication exposure is almost universal, their contribution to (or protection against) neuroenergetic vulnerability deserves examination.

The background vulnerability identified as Stage I of the hypothesis is not considered to cause FDS directly, but rather, is thought to reduce the buffer between normal cerebral oxygenation and the threshold at which acute failure can occur. In this way, triggers that would normally be tolerated by a sufficiently oxygenated brain can become enough to precipitate a seizure.

### 2.2 Stage II: acute metabolic crisis and network collapse

A trigger, whether it be a minor psychological stressor, an orthostatic challenge, a perceived threat, a sudden attentional demand, sensory overload, physical exertion, dysphoric rumination, or an intercurrent illness may produce a sudden surge in metabolic demand or a transient reduction in supply. In a brain with already depleted physiological reserve, this can drive the oxygen supply/demand balance into a deficit.

Under normal circumstances, the brain responds to falling oxygen levels by proactively reorganising neural networks (28). This proactive metabolic defence has been documented in neuroimaging studies which have found that functional connectivity within the default mode network (DMN) undergoes what Kang and colleagues (29) describe as ‘structured reorganisation’ under graded hypoxic challenge before detectable changes in peripheral oxygen saturation, which is consistent with a threshold-triggered rather than a gradually unfolding response (28, 30). This response is designed to preserve high-level functions. The DMN co-ordinates this reorganisation response, synchronising with the cognitive control and attentional networks. The threshold-triggered response is driven by physiology rather than expectation, prediction, or psychological state, and precedes any conscious awareness of oxygen insufficiency. Consistent with this, Kang and colleagues (29) report that in healthy adults this reorganisation fails at a reproducible threshold occurring at ~50% of individual baseline PetO_2_, with the protective window preceding behavioural impairment by ~66 s, a finding that would provide the most direct quantification of the Stage II mechanism if replicated. In people with FDS, the hypothesis advanced here is that this proactive reorganisation fails - a claim that has not yet been tested in FDS and which is an empirical target of one of the hypothesis’s central predictions. Instead of an orderly adaptive redistribution, brain networks start to fail, following the brain’s metabolic hierarchy (30). The reasons underlying the variable thresholds for reorganisation failure among FDS patients are likely multifactorial and cannot be attributed to a single cause. Chronic neuroinflammation may reduce network flexibility (31); white matter pathology documented in FDS neuroimaging can constrain the structural routes available for redistribution (32); mitochondrial dysfunction and impaired oxidative phosphorylation documented in neurodevelopmental conditions including autism spectrum disorder (ASD) and attention deficit hyperactivity disorder (ADHD) may reduce baseline metabolic reserve and compress the available margin (33–35); and cumulative depletion from prior FDS episodes lowers it further. The heterogeneity of FDS presentations may reflect, at least in part, the predominance of these mechanisms in any given individual.

Brain functional connectivity is organised along a principal gradient from transmodal, high-cost, highly connected hubs at one pole to unimodal, lower-cost sensorimotor and visual regions at the other (15). Transmodal regions, such as the DMN, prefrontal cortex, and anterior cingulate, carry substantially higher energetic signalling costs than unimodal regions (15). Within the DMN itself, posterior nodes including the precuneus and posterior cingulate are less metabolically costly than anterior prefrontal and cingulate nodes (15). Metabolic failure is not uniform across brain networks. The cognitive and awareness changes that precede or accompany the motor phase of FDS while elementary sensory function is at least partially preserved, are exactly what a cost-ordered collapse would produce, and how far down the gradient that collapse extends may be what distinguishes one FDS phenotype from another. For example, in patients who have predominantly dissociative presentations with minimal motor features, failure is hypothesised to reach the transmodal pole without extending into unimodal motor cortices, whereas for those with convulsive presentations, the failure extends further. More limited episodes are attributed to a partial failure or failure that is rapidly reversed. The gradient is a spectrum, not binary, with the depth of failure in any given episode determining semiology.

Preserved awareness with lost responsiveness appears to run counter to the gradient account. If motor control costs less than awareness, then awareness should fail first. However, responsiveness is not the same as awareness, and the distinction is important. Speaking and moving require high-overhead executive and motor integration networks. Knowing you are having an episode, tracking your surroundings, and hearing the people around you - these are sustained by posterior DMN nodes including the precuneus and posterior cingulate, which sit lower on the cost gradient than the anterior executive and motor integration networks that coordinate voluntary response. The gradient predicts that the expensive networks fail first (15). In these patients, the expensive ones are hypothesised to have failed first.

### 2.3 Stage III: the corrective motor response

When oxygen delivery to the brain falls critically low, the body drives blood back to the brain through skeletal muscle contraction, venous compression, and postural collapse (36). It is therefore argued that FDS motor symptoms may be physical expressions of what is going right, rather than what has gone wrong.

Tonic stiffening and postural collapse bring the brain to the level of the heart and the hydrostatic barrier to cerebral perfusion disappears. Rhythmic muscle contraction then compresses venous vessels and drives blood toward the thorax. From a haemodynamic standpoint, both effects operate in the same direction. These mechanisms are known to operate in other contexts of hypotensive threat (e.g., vasovagal pre-syncope response, motor automatisms in syncope recovery) (36, 37). Tonic stiffening of the lower limbs and trunk is simple in its mechanism, compressing the large venous capacitance vessels. While rhythmic jerking is less obvious in its effect, it is no less relevant, with each compression-release cycle advancing venous blood a little further toward the thorax. Opisthotonus induces sustained contractions of the paraspinal muscles by extending the spine and stiffening the core. From a purely mechanical standpoint, this pattern is difficult to distinguish from a deliberate Valsalva-adjacent effort. Fluctuating perfusion status, in which motor recruitment intensifies when perfusion falls and partially attenuates as it recovers, is predicted to produce the waxing and waning quality characteristic of some FDS episodes. Pelvic thrusting, often cited as an indication of FDS, involves large proximal muscle groups of a kind that, in other contexts, are effective activators of venous return. Ictal vocalisation engages the diaphragm and intercostal muscles, which in other contexts generate thoracic pressure changes that augment venous return. Whether these movements actually produce the haemodynamic effects their anatomy would predict, in the specific context of an FDS episode, has not been measured.

Rather than every FDS motor feature needing to be optimally haemodynamic, the hypothesis requires that the motor phenotype as a whole is more consistent with a perfusion rescue response than with random network disorganisation. Therefore, it is proposed that the motor phenotype of FDS is *functional*, rather than incidental. The existing motor automatisms of syncope recovery, which involve similar limb movements and are unambiguously physiological in their context (36), provide a precedent for considering whether the FDS motor phenotype might serve an analogous function. The analogy is suggestive but not evidentiary - syncope automatisms occur against a background of documented global cerebral hypoperfusion, and equivalent haemodynamic measurements during FDS do not yet exist. Whether the same mechanism operates is the question Prediction 1 is designed to test.

The Stage III hypothesis prediction is that cerebral blood flow velocity, measurable by transcranial Doppler, should increase during the motor phase of FDS, with recovery towards baseline concurrent with ictal motor activity rather than after motor activity has ceased. This prediction directly differentiates the neuroenergetic account from existing models. Stage III of the hypothesis may also explain why FDS episodes may sometimes be brief by predicting that successful motor rescue restores oxygenation within minutes. It is also proposed that FDS episodes terminate not because a psychological process is completed, but because the physiological perturbation is corrected. Incomplete or delayed rescue are proposed to predict prolonged FDS episodes, post-ictal confusion, and a slower recovery.

### 2.4 Stage IV: restoration and recovery

Suppressed networks are hypothesised to recover via a corrective motor response, spontaneous cardiovascular recovery, or external intervention as cerebral oxygenation normalises. By the same energetic logic that governs Stage II, the networks most sensitive to oxygen insufficiency are also the quickest to recover when supply is restored, consistent with the rapid cortical reoxygenation documented in controlled hypoxia studies (30). The DMN and prefrontal cortex return to function once oxygenation levels are restored, which explains the recovery of cognitive function (and consciousness for those with impaired awareness episodes). Studies using structured responsiveness testing (standardised verbal and command testing performed by epilepsy monitoring unit staff) during captured FDS events have described this pattern of preserved sensory processing with absent voluntary motor response as “ictal awareness with apparent unresponsiveness” (32)—the expected clinical correlate of partial gradient failure.

### 2.5 Stage V: trajectory divergence - a relapsing–remitting course

FDS follows a relapsing–remitting course in which a fluctuating threshold over time (rather than a fixed trajectory towards binary outcomes - recovery or chronification) determines episode burden at any given point. At any time, where the threshold sits is critical. If it sits above the seizure-triggering level, episodes do not occur. If it sits below, they do. The threshold position may be determined by whether the oxidative damage and neuroinflammatory load from each episode can be cleared before the next one arrives.

Some patients may deteriorate over time, potentially because episode burden consistently outpaces repair, not because chronification of the condition is inevitable. If damage from previous episodes does not dissipate before seizures recur, oxidative load may build, neuroinflammatory burden can accumulate, and consolidation of maladaptive network patterns may occur with each subsequent seizure. In this way, the threshold may further decrease, not necessarily permanently, but progressively, as long as the imbalance persists.

When repair keeps pace with episode burden, the threshold may recover, and episodes cease. As a result, the patient enters remission. The vulnerability that produced the episodes has been outweighed, rather than extinguished, and further accumulation of vulnerability factors can tip the balance back below the seizure-triggering level. Remission is considered a phase, not an endpoint.

The position of the threshold at any point in time (i.e., whether the patient is currently in a relapsing or remitting phase) may be determined by the balance between depletion and repair rather than by illness duration alone. Factors that might contribute to the trajectory include episode burden, baseline reserve, sleep architecture, iron status, physical fitness, allostatic load, and neuroplasticity. Children remit at high rates not simply because they are young, but potentially because their reserve has not yet been eroded by years of cumulative vulnerability and their neuroplasticity favours threshold recovery over threshold consolidation (38). The same logic applies to adults with short illness duration, low episode frequency, and intact physiological reserve.

The relapsing–remitting model can account for several clinical observations that have proved difficult to explain within existing frameworks. First, a proportion of patients substantially improve or spontaneously remit after receiving a diagnosis of FDS. Receiving a diagnosis may reduce anxiety-driven sympathetic nervous system activity and hypervigilance, both of which raise resting metabolic demand. In addition, following diagnosis, antiseizure medications are commonly withdrawn, and these medications have been shown to affect cerebrovascular tone and network energetics. For patients in whom the threshold has not yet drifted substantially below the triggering level, these concurrent changes may be sufficient for threshold recovery and entry into remission. Studies have documented seizure cessation in approximately half of patients who did not receive psychological therapy, and immediate remission following diagnosis alone in approximately one third of patients diagnosed in an epilepsy monitoring unit, consistent with the threshold recovery account once maintaining factors are removed (39, 40). Second, patients with longstanding FDS occasionally enter spontaneous remission - a pattern consistent with a relapsing–remitting course. This is predicted when life circumstances produce a sustained reduction in the vulnerability factor load, such as reduced allostatic burden, treatment of previously unrecognised sleep-disordered breathing, correction of iron deficiency, return to physical activity, or in women, the post-menopausal attenuation of hormonal amplifiers. Third, patients who have been in remission for years occasionally relapse, which is the defining feature of a relapsing–remitting condition. The NEDS model predicts that a relapse will coincide with renewed accumulation of vulnerability factors rather than a spontaneous occurrence, distinguishing neuroenergetic relapse from a purely psychological account in which relapse would be expected to follow psychosocial stressors without measurable physiological change.

Two patients with equivalent illness duration - duration being a crude proxy for the biological threshold state - could have entirely different cumulative bioenergetic burdens depending on the frequency of their episodes, as well as the cumulative load of vulnerability factors. This hypothesised mechanism is consistent with the documented association between longer illness duration and poorer prognosis (41, 42) and further predicts that this relationship should hold independently after controlling for comorbidity burden.

**Figure 1:**
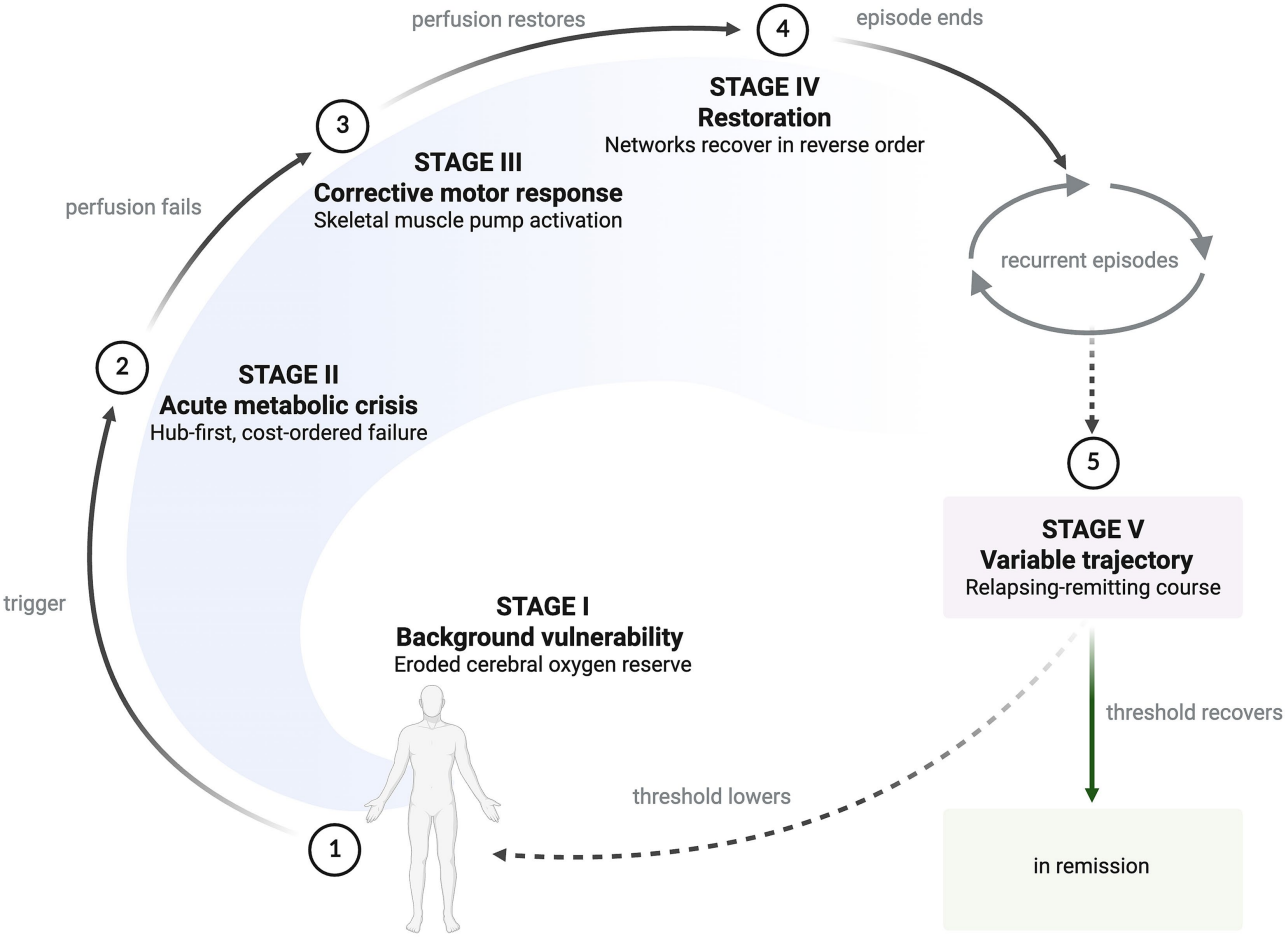
The neuroenergetic deficit syndrome: A five-stage mechanistic account of functional/dissociative seizures. (1) Background vulnerability. Multiple interacting pathways erode the buffer between normal cerebral oxygenation and the failure threshold. (2) Acute metabolic crisis. A trigger drives oxygen supply/demand balance into deficit; brain networks fail in hub-first, cost-ordered sequence from transmodal to unimodal regions. (3) Corrective motor response. Ictal motor activity activates the skeletal muscle pump, driving venous return and restoring cerebral perfusion. (4) Restoration. Oxygen delivery normalised, suppressed networks recover rapidly in reverse order of failure. (5) Variable trajectory. FDS follows a relapsing–remitting course. When repair keeps pace with episode burden the threshold recovers and the patient enters remission. When depletion outpaces repair the threshold drifts lower and episodes continue or worsen. The same patient may transition between these states across the lifespan depending on the balance between vulnerability factor load and bioenergetic repair capacity.

Stage V of the hypothesis also predicts that interventions that restore bioenergetic reserve (such as high-intensity exercise, treatment of anaemia, continuous positive airway pressure (CPAP) for OSA, and dietary modification) should help move the patient towards remission by raising the threshold above the level that might trigger a seizure. These interventions would complement existing psychological interventions by targeting a distinct mechanistic pathway. The proposed five-stage hypothesised mechanism is illustrated in Figure 1.

### 2.6 How the hypothesis differs from existing accounts

The neuroenergetic hypothesis of FDS distinguishes itself from existing accounts in several respects. First, it reframes the primary mechanism as physiological rather than psychological. Chronic stress, trauma, and anxiety are still relevant to the development of FDS. They may modulate oxygen reserve and network reorganisation capacity; however, modulating a threshold is not the same as causing the disorder, and the distinction matters for treatment. A patient whose dominant vulnerability is physiological will not be adequately served by psychological intervention alone. The female preponderance is handled differently here too. Where most accounts note the female preponderance and move on, this one asks why and finds the answer in sex-specific differences in hypoxic compensation rather than solely in demographic observation. The differences in hypoxic compensation between males and females are measurable, emerge at puberty, and attenuate after menopause. The female preponderance is predicted here from physiology rather than being accommodated to fit an observed pattern. It is important to note that sex-specific hypoxic physiology is offered as a candidate contributing mechanism, and does not exclude the influence of sociocultural, developmental, and psychiatric factors, which also likely interact to produce the observed female-to-male ratio.

The normal ictal EEG in FDS is consistent with the neuroenergetic hypothesis rather than contradictory to it. The hypothesis does not predict epileptiform electrical discharges - it predicts cerebrovascular, metabolic, and network reorganisation changes that are visible on functional near-infrared spectroscopy (fNIRS), transcranial Doppler (TCD), and magnetic resonance spectroscopy (MRS) but not on standard scalp EEG. The EEG correctly identifies the absence of epileptiform activity; it does not capture the haemodynamic and metabolic substrate the hypothesis proposes. Quantitative EEG evidence of altered interictal complexity and pre-ictal entropy change is consistent with but does not confirm the mechanism - for the same reason that an absence of ictal epileptiform activity does not refute it. Both observations reflect the limits of what scalp EEG measures, not the presence or absence of a neuroenergetic mechanism.

A common observation is that FDS motor features resemble movements a person could produce voluntarily, unlike epileptic seizures or syncopal events. This does not distinguish between a psychological conversion account and a physiological rescue account. The skeletal muscle pump activation during Stage III may recruit the same motor pathways as voluntary movement because both serve the same anatomical function - venous return and postural control. The motor automatisms of syncope recovery are similarly voluntary in appearance yet unambiguously physiological. The discriminating question is not whether the movements are possible voluntarily, but whether they are accompanied by the haemodynamic changes Stage III predicts - a question directly testable by concurrent transcranial Doppler monitoring and not yet examined.

It is important to note that the neuroenergetic hypothesis does not require an organic trigger. Psychological stressors, perceived threat, emotional arousal, rumination, and/or sudden attentional demand are all potential triggers in Stage II of the model, consistent with the well-documented association between psychological precipitants and FDS. The distinction the hypothesis draws is not between organic and psychological triggers, but between the trigger, which can be psychological, and the physiological cascade that follows the threshold breach, which is proposed to be neuroenergetic rather than conversion-based. A psychological trigger that occurs against a background of a narrowed neuroenergetic threshold is entirely consistent with the biopsychosocial framework and provides a more mechanistically complete account of *how psychological states produce physical symptoms* than conversion theory alone.

The finding that FDS and functional motor disorder (FMD) show distinct psychiatric comorbidity profiles in a large-scale electronic health record analysis of over 120,000 cases is consistent with the neuroenergetic account (43). Depression, anxiety, post-traumatic stress disorder (PTSD), and substance use are more common in FDS, whereas somatoform disorders are more common in FMD. The psychiatric conditions overrepresented in FDS are those most strongly associated with dysfunctional breathing patterns, chronic sympathetic hyperactivation, and HPA axis dysregulation - the biological pathways through which psychological vulnerability can lower the neuroenergetic threshold. This differential psychiatric loading supports a mechanism specific to FDS that operates through breathing and autonomic dysregulation. Finally, the hypothesis provides a functional account of FDS phenomenology. Existing accounts treat motor symptoms as disorganisation (32) or freeze (44). The neuroenergetic account treats them as rescue. The difference matters clinically. It may change how symptoms are explained to patients and what interventions are designed to do.

#### 2.6.1 Distinction from vasovagal syncope

Vasovagal syncope involves haemodynamic collapse with a loss of postural tone and brief motor automatisms (36). The superficial similarity to some FDS clinical presentations may raise the question of whether the neuroenergetic mechanism is describing a variant of syncope. The clinical duration of FDS episodes substantially exceeds that of typical vasovagal episodes and FDS frequently occur without orthostatic provocation, which suggests a distinct yet related mechanism (36, 37). These distinctions between FDS and vasovagal syncope are hypothesis predictions, which require formal testing via haemodynamic comparison.

The distinction from syncope runs deeper than it might appear. Vasovagal hypoperfusion is global and rapid - all networks go down together, which is why there is no gradient-ordered progression and why consciousness is lost all at once rather than in stages. Repeated episodes of syncope do not appear to lower their own threshold for further events - collapse is brief, reperfusion is typically rapid, and the cumulative pattern characteristic of FDS has not been documented in syncope. Pre-syncope produces a uniform prodrome (visual greying and generalised weakness) because all of the networks are affected simultaneously. These prodromal symptoms signal early global hypoperfusion, not the heterogeneous symptom profile of cost-ordered network failure. Further, because FDS are not orthostatic in nature, postural collapse in and of itself does not resolve them. Active muscle contraction, not postural change, restores network perfusion - which is why the motor phase is hypothesised to last as long as it needs to. This is also why Stage III may be mechanistically necessary rather than incidental. A related question is whether the two conditions affect the blood–brain barrier similarly. In a case study by Huh and colleagues (45), vasovagal syncope produced measurable blood–brain barrier (BBB) disruption on dynamic contrast-enhanced MRI (DCE-MRI) after a single brief episode, with the proposed mechanism being ATP depletion from transient cerebral hypoxia leading to endothelial dysfunction. This cellular cascade is the same one that the neuroenergetic hypothesis proposes for FDS, suggesting that BBB disruption may also be involved in FDS, but with a different temporal and spatial signature. Because FDS episodes are repeated, the prediction is that of cumulative BBB stress over time rather than the single-episode pattern documented in syncope. FDS hypoperfusion is proposed to be network-selective rather than global, so BBB changes are predicted to appear in specific networks rather than diffusely across the cortex. DCE-MRI permeability mapping is the relevant test that could be compared across FDS patients, matched syncope cohorts, and healthy controls.

### 2.7 Evidence in support of the hypothesis

Before reviewing the evidence, an important distinction. The evidence cited below (particularly the symptom-overlap evidence in Section 2.7.1) comes predominantly from systemic hypoxia paradigms (altitude chamber, aviation, reduced inspired oxygen). In these studies, cerebral oxygen insufficiency is produced via whole-body desaturation rather than through the localised cerebrovascular supply–demand mismatch the hypothesis proposes. What these studies establish is that the FDS symptom catalogue can be reproduced by cerebral oxygen insufficiency of any cause. They do not establish that the specific mechanism proposed here (i.e., local rather than systemic insufficiency) actually operates in FDS. That is what Prediction 1 is designed to test.

#### 2.7.1 The overlap between symptom profile and cerebral hypoxia

Clinical symptoms of FDS overlap with the documented physiological effects of cerebral oxygen insufficiency. Common FDS presentations include altered consciousness (sudden loss of awareness/blank spells), abnormal movements (jerking, twitching, muscle stiffening), respiratory changes (shallow or rapid breathing/hyperventilation, breath holding), autonomic features (tachycardia, diaphoresis, pallor), cognitive symptoms (confusion, dizziness, concentration difficulties/brain fog), and convulsions (46). This suite of symptoms is also the symptom catalogue of controlled cerebral hypoxia, with experimental induction of graded hypoxia in healthy people without psychological vulnerability exhibiting the same symptom profile (13, 36, 47, 48). This suggests that while psychological vulnerability is a threshold lowering factor, it is not a necessary condition for FDS. Further, oxygen desaturation occurs during FDS at rates comparable to epileptic seizures, with breath holding after a period of hyperventilation posited as a plausible mechanism in some cases (49). The recent discovery of localised hypoxic pockets in the cortical tissue of mice, where transient areas of oxygen depletion within circumscribed cortical regions occur even in healthy brains when oxygen delivery fails to meet metabolic needs (particularly during intense neural activity or capillary flow disruption) (50), suggests that focal metabolic insufficiency may be a normal feature of brain physiology that becomes pathological when there is inadequate reserve. This evidence provides a potential cellular mechanism for the focal-onset features observed in some FDS presentations.

The well-documented tendency of FDS patients to hyperventilate, established at the meta-analytic level, requires engagement within the neuroenergetic framework. A study of hyperventilation patterns in FDS identified distinct subtypes - including one without hyperventilation, which means that the hyperventilation evidence cannot be applied universally across all FDS presentations (51). In the hyperventilation subtype, hypocapnia-driven cerebrovascular vasoconstriction, via CO_2_ reactivity, can worsen cerebral oxygen delivery despite normal or elevated peripheral saturation (19). Pre-ictal hyperventilation driven by incipient cerebral oxygen insufficiency produces hypocapnia, which constricts cerebral vessels, which worsens the insufficiency it was trying to correct. The attempt at compensation becomes part of the problem. It has been suggested that the hyperventilation account predicts the opposite direction of oxygen change to the neuroenergetic hypothesis since hyperventilation raises peripheral oxygen saturation. This apparent contradiction dissolves once the cerebral versus peripheral distinction is applied. Hypocapnia constricts cerebral vessels. That is the CO_2_ reactivity mechanism - well established and not contested (19). Hyperventilation produces hypocapnia. So a patient who hyperventilates before a seizure may be raising peripheral oxygen saturation while simultaneously cutting cerebral oxygen delivery. The two accounts are not pointing in opposite directions. They are pointing at the same endpoint via different paths. The hyperventilation evidence does not contradict the neuroenergetic hypothesis. The hyperventilation subtype represents one physiological pathway to the same cerebral insufficiency that the hypothesis proposes, while the non-hyperventilating subtype represents another.

This raises a clinical question of whether hyperventilation monitoring could serve as a diagnostic or predictive tool. Provocation-based PCO_2_ measurement during hyperventilation challenge is already established in both epilepsy and FDS, and has demonstrated distinct CO_2_ trajectories in FDS patients compared to controls (52). What has not been characterised is the pre-ictal CO_2_ signature in everyday ambulatory contexts, where continuous capnography in this population may be limited by motion artefact during episodes. Whether transcutaneous CO_2_ monitors, accelerometer-corrected capnography, or short-window sampling around motion-free intervals could resolve this limitation is a methodological question for prospective ambulatory studies. This prediction relates to the hyperventilation subtype identified by Kanaan and colleagues (51)—the non-hyperventilating subtype is not expected to show this signature.

Independent convergent evidence comes from within the paediatric FDS literature. Kozlowska and colleagues identified a non-hyperventilation subgroup in which FDS occur because psychological distress triggers airway occlusion or defensive vagal activation, resulting in decreased cerebral blood flow, explicitly describing an overlap between what neurologists categorise as physiological and “psychogenic” non-epileptic seizures (53). The hyperventilating subgroup brings the accounts into direct alignment. Prolonged hyperventilation drops CO_2_, constricts cerebral arteries, and produces hypoxia sufficient to disconnect the cortex from lower brain structures. That is Stage II - described here in neuroenergetic terms, described there in neurophysiological ones. Further, symptoms of cerebral hypoxia were almost five times more frequent in FDS patients than controls during a hyperventilation challenge (52), providing direct empirical support for cerebral oxygen insufficiency as a mechanism in FDS.

The most direct challenge to the neuroenergetic hypothesis is the evidence that FDS patients are generally well oxygenated peripherally during a seizure. Studies measuring peripheral oxygen saturation during captured FDS episodes have not consistently demonstrated ictal desaturation. For example, one study found desaturation in approximately a quarter of events; however, in the remaining episodes, patients were well saturated (49). Although this finding does not refute the hypothesis, it requires the hypothesis to rest entirely on *cerebral* oxygen insufficiency rather than *peripheral* oxygen insufficiency - a distinction that is mechanistically coherent but currently lacks direct haemodynamic confirmation during FDS episodes. The central prediction of the hypothesis - that cerebral rather than peripheral oxygenation is reduced in the pre-ictal and ictal period - remains the primary empirical target and has not yet been directly tested.

#### 2.7.2 Sex preponderance from physiological first principles

FDS affect three times as many females as males in adulthood, however, this ratio is not stable across the lifespan. In pre-pubertal children, the female-to-male ratio approaches parity and increases gradually through adolescence and adulthood with a difference between pre-pubertal and adult populations before attenuating after menopause (12). This pattern suggests that reproductive hormonal physiology may play a role in FDS, rather than trauma exposure or socialisation (neither of which are confined to the reproductive years or show puberty-onset and menopause-attenuation). The neuroenergetic hypothesis derives this pattern from known sex differences in hypoxic physiology. Oliveira and colleagues (54) found that under hypoxic challenge, males primarily deploy ventilatory compensations - increasing tidal volume and decreasing breathing frequency - to restore arterial oxygenation. In contrast, females deploy circulatory compensation exclusively, marked tachycardia and increased cardiac output, without any ventilatory adjustment (54). When the female circulatory defence is impaired, there is no ventilatory backup available. As females in this study were assessed during the low-progesterone follicular phase, the circulatory-ventilatory difference observed likely represents a conservative estimate - the sex difference the neuroenergetic hypothesis proposes is amplified during the luteal phase and in the early post-pubertal years when progesterone levels are highest.

Direct microneurography measurement has demonstrated that under combined central and peripheral chemoreflex activation, women show augmented total muscle sympathetic nerve activity (MSNA) with attenuated ventilatory responses (20). This sex-specific compensatory pattern means that conditions that impair the circulatory defense such as anaemia (via a reduction in oxygen carrying capacity) (16), progesterone (by simultaneously increasing ventilatory sensitivity and causing cerebrovascular vasoconstriction) (55), or impaired sympatholysis in obese women (by reducing vasodilatory relief during hypoxia) (21) can compromise the female compensatory mechanism. The circulatory dominant female compensatory strategy emerges with post-pubertal hormonal cycles. Prior to the onset of puberty, sex differences in hypoxic compensation are minimal (12), which may explain the near-parity female-to-male ratio in paediatric FDS. After menopause, progesterone-driven cerebrovascular vasoconstriction and associated hypocapnic burden decrease (55), which reduces the specific physiological vulnerabilities that may amplify the mechanism during the reproductive years.

The strongly post-puberty onset distribution of FDS with a modal age of onset of 23 years confirmed in a large-scale electronic health record analysis of over 120,000 cases (43) is consistent with the reproductive hormonal amplifier account proposed here, in which the physiological changes of puberty narrow the neuroenergetic margin specifically in females through progesterone-driven ventilatory sensitisation and sex-specific differences in hypoxic compensation (54, 55).

#### 2.7.3 Neurometabolic evidence

Neuroimaging studies have provided preliminary evidence of bioenergetic dysfunction in FDS populations. For example, MRS studies have documented reduced N-acetylaspartate to creatine (NAA/Cr) ratios (a marker of neuronal metabolic function) in the dorsomedial prefrontal cortex, anterior cingulate cortex, and thalamus in adults with FDS (56). NAA is synthesised primarily in neuronal mitochondria and its reduction reflects compromised mitochondrial oxidative metabolism (57). Higher myo-inositol, a marker of glial activation consistent with neuroinflammatory response, has been found in the precentral gyrus, posterior temporal gyrus, anterior cingulate, and orbitofrontal cortex in FDS patients compared to healthy controls (31). While these preliminary findings require replication with medication-controlled study designs, they are directionally consistent with the neuroenergetic account. In addition, functional neurological disorder (FND) more broadly has been characterised by dysregulated bioenergetics and increased vulnerability to oxidative stress in a neurometabolic network analysis (58). Neurometabolic dysfunction appears to extend across FND presentations, though direct replication in adults with FDS is needed. The metabolic cost of network reorganisation may be directly relevant, given that it is one of the most energetically demanding brain processes. If bioenergetic reserve is depleted, the proactive reorganisation response that normally protects against hypoxic failure may not be available.

#### 2.7.4 Paediatric prognosis

The prognosis of FDS in children is substantially better than in adults. Combined seizure remission or improvement is 89% at 12-month follow up in paediatric cohorts (38). Younger age at diagnosis independently predicts FDS improvement after controlling for illness duration and other confounders (38). The neuroenergetic hypothesis directly predicts this pattern. Children are typically diagnosed earlier in the process (5, 38). The physiological hyperarousal documented in paediatric FDS cohorts (i.e., elevated heart rates associated with seizure frequency) (59) reflects acute sympathetic loading rather than exhausted reserve. What protects children may not be age itself but the biological conditions that tend to accompany it; that is, intact bioenergetic reserve, lower cumulative episode burden, higher neuroplasticity, and the absence of post-pubertal hormonal amplifiers that narrow the margin in adolescent girls and women of reproductive age (38). The same conditions predict remission in adults. Short illness duration, low episode frequency, and no established neuroenergetic depletion markers may be the adult equivalents of the paediatric advantage.

#### 2.7.5 Treatment response pattern

The landmark CODES (Cognitive Outcomes and adverse Events from Dissociative Seizures) trial results, which found that cognitive behavioural therapy (CBT) demonstrated greater benefit in patients with high psychiatric and somatic comorbidity while not reducing FDS frequency at 12-months in the unselected FDS population (14, 60), are what the neuroenergetic hypothesis predicts. This does not dismiss CBT: rumination, avoidance, and emotional dysregulation modulate the threshold, and must be addressed. CBT, however, may not restore cerebral oxygen delivery or network reorganisation capacity, and for patients whose vulnerability is primarily physiological rather than psychological, that may be what needs restoring. The results of the moderation analysis of the CODES study (i.e., CBT worked better in patients with a higher burden of psychiatric comorbidity) potentially identifies a subgroup of FDS patients whose vulnerability is predominantly psychological threshold-loading rather than physiological background vulnerability. Matching treatment allocation to mechanism by offering more targeted physiologically relevant interventions to those with identifiable oxygen delivery deficits, and psychologically relevant interventions for those with predominantly psychological loading, is a prediction generated by the current hypothesis and which the CODES findings support. Consistent with this prediction, interventions that target specific physiological vulnerabilities are supportive of the current hypothesis. For example, breathing retraining in people with documented hyperventilation has found a correlation between Nijmegen hyperventilation score and FDS frequency reduction (61); treating OSA with CPAP has produced measurable recovery in prefrontal executive function and grey matter volume (17); and iron correction in iron-deficient women has demonstrated an up to fivefold improvement in cognitive performance accuracy (62).

### 2.8 Discriminating hypothesis predictions

The neuroenergetic hypothesis generates the following predictions to distinguish it from existing accounts.

#### 2.8.1 Prediction 1: ictal cerebral blood flow

Cerebral blood flow velocity measured by transcranial Doppler will increase during the motor phase of FDS relative to the pre-ictal period. This prediction directly discriminates the neuroenergetic account from psychological conversion accounts since no haemodynamic change concurrent with ictal motor activity would be predicted by a purely conversion-based mechanism. Sustained or increasing hypoperfusion during ictal motor activity would refute Stage III of the hypothesis.

Hyperperfusion in the right ventromedial prefrontal cortex (vmPFC) and right insula was identified in a preliminary SPECT study of ictal cerebral blood flow during FDS events, with no regions of hypoperfusion (63). Within the neuroenergetic framework, ictal vmPFC hyperperfusion is not straightforwardly compatible with the hypoperfusion proposed in Stage II of the hypothesis, however, given that the tracer was injected up to 125 s after seizure onset, the scans may have captured Stage III or Stage IV rather than Stage II. The small sample of six patients, the heterogeneous population including three with comorbid temporal lobe epilepsy, and the absence of pre-ictal measurements limit the conclusions that can be drawn. The discriminating prediction of the neuroenergetic hypothesis - that pre-ictal and early ictal cerebral oxygenation is reduced relative to interictal baseline - has not been directly tested by existing SPECT methodology, which captures a single time point and cannot resolve the dynamic sequence the hypothesis proposes.

#### 2.8.2 Prediction 2: hypoxic challenge discrimination

FDS patients will show earlier failure of the proactive DMN reorganisation response under graded hypoxic challenge compared to matched controls and matched patients with equivalent psychiatric and somatic comorbidity without seizures. This prediction would distinguish FDS from general allostatic load vulnerability accounts. Equivalent DMN reorganisation capacity under hypoxic challenge in FDS and controls with comorbid psychiatric and somatic burden would undermine the mechanism.

#### 2.8.3 Prediction 3: pre-ictal cerebral oxygenation

If Stage II of the hypothesis is correct, one would expect to see reduced cerebral oxygenation in the pre-ictal period relative to matched interictal baseline levels and steeper drops in cerebral oxygenation prior to the onset of more severe FDS episodes. Normal pre-ictal cerebral oxygenation would be difficult to reconcile with the proposed mechanism.

#### 2.8.4 Prediction 4: sex-stratified hypoxic compensation

FDS vulnerability will be predicted by markers of impaired circulatory compensation to hypoxic challenge in women (i.e., reduced heart rate response, impaired sympathetic activation) rather than by impaired ventilatory compensation. FDS vulnerable women will show a specific profile or circulatory defence failure under standardised hypoxic challenge. Absence of sex-specific differences in the hypoxic compensation profile of FDS patients would undermine the sex difference mechanism.

#### 2.8.5 Prediction 5: condition-matched treatment

In stratified trials, physiological intervention (e.g., iron supplementation in anaemic patients, CPAP in OSA, breathing retraining in documented hyperventilators, aerobic exercise) will reduce FDS frequency in patients with identified physiological vulnerability that is not achieved by psychological intervention alone in these same patients. Psychological interventions will produce greater benefit in patients without identified physiological vulnerabilities. Equivalent responses to psychological intervention regardless of the physiological vulnerability profile would challenge the mechanism-treatment mismatch prediction.

#### 2.8.6 Prediction 6: biomarker profile

Two findings are predicted, and one non-finding. Cerebrovascular CO_2_ reactivity will be impaired in FDS patients relative to somatic symptom controls with equivalent psychiatric comorbidity. Anterior cingulate NAA/Cr will be reduced. General oxidative stress and inflammatory markers will not differ between groups. This specific pattern of difference and non-difference is what the neuroenergetic account predicts and what a general allostatic load account does not.

#### 2.8.7 Prediction 7: biomarkers will predict relapsing–remitting phase better than illness duration

Regardless of illness duration, cerebrovascular CO_2_ reactivity, resting prefrontal fNIRS oxygenation, and anterior cingulate NAA/Cr should predict treatment response and illness trajectory. The falsifying condition is that there will be no relationship between biomarker state and trajectory after controlling for duration.

## 3 Evidence against the hypothesis and limitations

There are evidential and conceptual challenges associated with this hypothesis that must be acknowledged. The populations in which the hypothesis predicts FDS should be overrepresented - for example, those with diagnoses of iron deficiency, OSA, and obesity - are already captured in large primary care databases, and a record-linkage study could test the prediction without requiring new data collection. No epidemiological study has been designed with this question in mind, and this represents a significant evidential gap. It is not evidence against the hypothesis. It is evidence that the hypothesis has not yet been put to the relevant test. One partial exception is the finding of moderate-to-severe sleep-disordered breathing in 29% of FDS patients (22), with the affected subgroup described by the authors as “often female and obese” - consistent with the hypothesis prediction that physiological vulnerability factors, including obesity-related respiratory limitation, will be overrepresented in FDS populations. This study, however, was not designed to test this prediction and lacked a healthy control comparison, so whether this rate exceeds population norms in age-matched women is not known.

The Kang finding (29) on which the predictive DMN reorganisation account rests, including the threshold mechanism and the ~66 s protective window, is from a sample of healthy adults and has not been replicated in clinical populations. The threshold and protective window figures should therefore be understood as provisional estimates pending independent replication, and as such, Stage II of the hypothesis rests on the broader evidence for DMN reorganisation under hypoxic challenge (28, 30) rather than on the specific quantitative parameters from a single source. Another challenge is the failure of supplementary oxygen administered during or prior to FDS episodes to consistently terminate them. If cerebral oxygen insufficiency does drive the mechanism, nasal or mask oxygen should, in principle, abort episodes. The fact that supplementary oxygen does not reliably terminate FDS episodes is consistent with the bioenergetic mechanism rather than contrary to it. The trigger for FDS is a local supply–demand mismatch - metabolic demand outpacing cerebrovascular delivery at the network level - not a deficit in inspired oxygen fraction. Normobaric supplementation at standard flow rates does not meaningfully raise dissolved plasma oxygen, and it does not address the autoregulatory failure that the mechanism proposes. Second, by the time supplementary oxygen is administered, Stage III of the hypothesised mechanism has typically been triggered already, and the motor rescue response is underway; terminating it would require restoring cerebral oxygenation faster than the episode’s own haemodynamic rescue mechanism, which normobaric supplementation cannot achieve. With regards to Stage III, while functional MRI has captured activation and deactivation patterns consistent with a dissociative event during a spontaneous episode of FDS in a case study, simultaneous haemodynamic measurements sufficient to test Stage III of the current hypothesis were not obtained (64), thus the central prediction of Stage III is unconfirmed.

The specificity of the condition - why FDS affects only ~0.1% of the population (1) when its proposed vulnerability factors are individually common - is one of the central open questions for any FDS account. The neuroenergetic hypothesis does not resolve it but does generate testable predictions about the structure of the answer. These vulnerability factors - anaemia, OSA, obesity, dysfunctional breathing, chronic stress - are individually common. If these factors lower the threshold, why do most people with these vulnerabilities never develop FDS? The hypothesis proposes that multiple vulnerability factors must be present to lower the reserve below a critical threshold, in a person whose cerebrovascular and network architecture produces network-selective rather than global hypoperfusion at threshold breach, and whose response is gradient-ordered failure and motor rescue rather than syncope, panic, or no clinical event at all. Each of these conditions is individually common - their conjunction is rare. The hypothesis does not yet specify which combinations of vulnerability factors are sufficient, how the combinatorial threshold scales with severity across them, or which features of network architecture lead to an FDS phenotype rather than to syncope or panic at threshold breach. These are the empirical questions that Section 3.1 is designed to address - particularly the sex-stratified hypoxic challenge studies (Prediction 4) and the biomarker profiling work (Predictions 6 and 7), which together aim to characterise the threshold-burden profile that separates FDS-vulnerable individuals from those who carry the same risk factors without developing seizures.

Similarly, the determinants of a patient’s relapsing–remitting course and more specifically, what drives transitions between relapsing and remitting phases following the onset of FDS, remain empirically unresolved. The hypothesis proposes that episode burden, baseline bioenergetic reserve, and repair capacity are the primary determinants rather than illness duration. The latter is a widely used but mechanistically indirect proxy. The evidence that illness duration does not reliably predict treatment outcomes (41, 42) is consistent with this account. For example, two patients with the same illness duration might differ substantially in cumulative bioenergetic burden depending on their episode frequency and their ongoing vulnerability factor profile. It is worth noting that the specificity challenge is not unique to the neuroenergetic account. Psychological vulnerability factors - anxiety, depression, PTSD, childhood trauma - are each far more prevalent in the general population than FDS itself, yet existing accounts do not resolve why these common vulnerabilities produce FDS in some individuals and not others. The specificity problem is an open question across the field, and the neuroenergetic account is not at a disadvantage relative to competing frameworks on this point.

The neurometabolic evidence for the hypothesis is cross-sectional and heterogeneous across the paediatric and adult literature as well as potentially confounded by medication use (31, 56). Antidepressants, anticonvulsants, and benzodiazepines have documented effects on NAA and myo-inositol (57). Therefore, the MRS evidence is suggestive rather than confirmatory until medication- controlled replication evidence is available. Given that rates of depression, anxiety, and PTSD in FDS populations substantially exceed those in other FND presentations, and that most of these patients will be on psychotropic medication at the time of neuroimaging, medication confounding of MRS bioenergetic markers is a systematic rather than incidental limitation. Medication-naïve or medication-washout study designs are necessary to establish whether the neurometabolic differences observed reflect the proposed mechanism or its pharmacological treatment.

As noted in Section 2.7, the nature of the supporting evidence for the core mechanism represents a further limitation. The hypothesis proposes that cerebral oxygen insufficiency with normal or near-normal peripheral saturation (a stagnant rather than hypoxic hypoxia mechanism) is driven by a supply–demand mismatch at the network level rather than by systemic desaturation. The majority of the evidence cited in support of the symptom profile and network failure sequence derives from systemic hypoxia studies (altitude chamber, aviation, and reduced inspired oxygen paradigms) in which cerebral insufficiency is produced via systemic desaturation rather than through the localised cerebrovascular mechanism proposed by the hypothesis. These studies establish that the FDS symptom catalogue is reproducible by cerebral oxygen insufficiency of any cause, but they do not confirm the specific mechanism proposed here. Direct evidence from continuous cerebral oxygenation monitoring using fNIRS or TCD across the pre-ictal, ictal, and post-ictal periods in FDS patients does not yet exist. This is the evidential gap that Prediction 1 of the hypothesis is designed to close. Indirect support comes from adjacent populations that share overlapping physiological profiles. For example, interictal reduced prefrontal oxygenation has been documented by fNIRS in migraine patients with aura during cognitive task performance, with normal peripheral saturation (65); and impaired prefrontal cerebral oxygenation under physiological challenge has been documented in chronic fatigue syndrome (66) - conditions that share cerebrovascular dysregulation, autonomic dysfunction, and fatigue with FDS.

The higher rates of psychiatric comorbidity in FDS relative to other FND presentations (43) raise the question of whether the neuroenergetic account adequately explains the specificity of this psychiatric loading. The hypothesis treats psychological vulnerability factors such as PTSD, anxiety, and depression as biological vulnerability factors that reduce the neuroenergetic margin through mechanisms like sympathetic hyperactivation, HPA dysregulation, and dysfunctional breathing - not as psychological causes or mechanisms alone. Whether this account fully explains why FDS carries a higher psychiatric burden than other FND presentations specifically remains an open empirical question. It is also worth noting that the authors of the large-scale study themselves caution that the temporal relationship between psychiatric comorbidity and FDS onset is difficult to establish from administrative records, and that the effect sizes, while statistically significant in a very large sample, are predominantly small (43).

Another question relates to the age distribution of FDS. If cumulative bioenergetic depletion lowers the threshold for FDS, then we might expect to see an increased prevalence of the condition with advancing age as networks degrade and reserve is eroded. The epidemiological pattern is the opposite. The hypothesis accounts for this as follows: the mechanism requires a specific configuration - reserve lowered but not yet exhausted, with selective rather than global network vulnerability. Diffuse cerebrovascular compromise is not the same thing as selective network-level oxygen insufficiency, and advancing age tends to produce the former rather than the latter. In addition, the reproductive hormonal amplifiers that narrow the margin in younger women disappear with age, making the configuration that the mechanism requires harder to sustain rather than easier. The clinical expression of network degradation in older age is more likely to be cognitive impairment, autonomic failure, or cerebrovascular disease than FDS. Whether this account survives epidemiological scrutiny has not been tested directly and represents a falsifiable prediction of the hypothesis (12).

The hypothesis must also account for patients with FDS who have no identifiable physical vulnerability on assessment. Several mechanisms might operate in this subgroup, including unmeasured cerebrovascular dysregulation, network architecture vulnerabilities not captured by current clinical workup, or the pseudohypoxic cellular environment produced by chronic stress and sympathetic nervous system activation (23). While the latter is common, it may render the hypothesis unfalsifiable at the individual level if used as a default explanation for any patient who lacks measurable physical vulnerability. This risk is avoidable given that the pseudohypoxic burden is measurable. HIF-1α and its downstream targets, reactive oxygen species, and chronic sympathetic loading can all be quantified. If the pseudohypoxic account is correct, patients who present without any identifiable physical vulnerability should still show elevations on these markers relative to matched controls. If they do not, the pseudohypoxic account is wrong (which means it fails on its own terms rather than absorbing every unexplained case).

Finally, the hypothesis presented is specific to FDS and does not claim to account for the full spectrum of FND presentations. Functional movement disorders, functional cognitive disorder, and persistent perceptual postural dizziness have different symptom profiles, may have different triggers, and different clinical trajectories. The observation that FDS frequently co-occurs with other FND presentations (3, 5) is not directly explained by the current formulation of the neuroenergetic mechanism. Whether a shared neuroenergetic substrate underlies the broader FND spectrum, or whether the co-occurrence reflects shared psychological and neurobiological vulnerability factors operating through different mechanisms is a question for the framework paper that this hypothesis is intended to generate rather than one it can answer. The current account is intentionally specific: a hypothesis about FDS that generates testable predictions is more scientifically useful than a unified account of FND that generates none.

### 3.1 Testing the hypothesis

#### 3.1.1 Neuroimaging and physiological studies

To directly test Stage III of the hypothesis, simultaneous transcranial Doppler and video-EEG monitoring during FDS could measure middle cerebral artery flow velocity concurrent with ictal motor activity. A test of Stage II failure could involve a graded hypoxic challenge using concurrent fNIRS and cerebrovascular reactivity measurement to compare those with FDS and those with somatic disorders without seizures. Ambulatory fNIRS in prospective FDS cohorts could be used to characterise pre-ictal oxygenation patterns and identify potential physiological trigger signatures. Finally, medication-controlled MRS studies of anterior cingulate bioenergetics that compare FDS to somatic disorder controls as well as healthy controls would be informative.

Quantitative EEG analysis offers a further and immediately tractable testing avenue. Interictal EEG complexity and alpha-band connectivity have been shown to differ between FDS and epilepsy patients in studies designed for diagnostic classification, with the number of FDS episodes per month correlating with disrupted local alpha connectivity, and entropy measures showing a significant drop in the pre-ictal period relative to interictal baseline (67, 68). These findings were not designed to test the neuroenergetic hypothesis but are consistent with it - a brain operating with narrowed bioenergetic reserve would be expected to show reduced electrophysiological complexity at rest and a steeper pre-ictal entropy decline as the threshold is approached. Both studies were conducted in clinically representative populations where medication use was not controlled, and the confounding effects of antidepressants and antiepileptic drugs on spectral power and connectivity measures cannot be excluded. Medication-naive designs or within-subject pre-post analyses would be needed to establish whether the EEG signatures reflect the underlying mechanism or its pharmacological treatment. Importantly, the critical experiment these papers make feasible but have not yet attempted is correlating the interictal EEG signature with physiological vulnerability measures. Whether FDS patients with anaemia, OSA, or documented dysfunctional breathing show greater pre-ictal entropy drops or greater alpha disruption than FDS patients without those vulnerabilities has not been examined and would constitute a low-cost, non-invasive test of the Stage I vulnerability account.

#### 3.1.2 Clinical intervention studies

The interventions outlined below fall into two categories. The first comprises established interventions with existing clinical indications that can also serve as tests of the mechanism when paired with mechanism-relevant endpoints. The second comprises investigational interventions derived from the hypothesis itself, which require trial-level mechanistic evidence before they are considered potential clinical interventions in FDS.

*Established interventions with mechanistic endpoints.* Aerobic training is indicated across most adult populations on cardiometabolic and mental health grounds. Within the neuroenergetic framework, it is proposed to act on cerebrovascular reserve and network flexibility, with fNIRS prefrontal oxygenation serving as the mechanistic endpoint (69). Iron supplementation in iron-deficient women with FDS could test the most specific and measurable physiological vulnerability pathway. CPAP is already indicated when OSA is confirmed, and breathing retraining when hyperventilation is documented, but what remains untested is whether either of these reduces FDS frequency through the mechanism proposed by the hypothesis.

*Investigational interventions derived from the hypothesis.* These are tests of the mechanism, not treatment recommendations. The trial-level evidence that would justify recommending any of them in FDS does not yet exist. Intermittent hypoxia training may target something more specific - the reorganisation capacity that repeated uncontrolled hypoxic episodes have degraded - by replacing uncontrolled events with controlled adaptive exposure (70, 71). A ketogenic diet trial could be used to test alternative metabolic substrate provision with MRS bioenergetics as a primary mechanistic endpoint (72, 73). In addition, exogenous ketone supplementation could be used to test acute metabolic rescue in the peri-ictal period (72). Hyperbaric oxygen therapy (HBOT) studies could assess whether increasing available oxygen reduces FDS frequency (74).

Designing mechanism-matched treatment allocation studies (e.g., stratified randomised controlled trials that assign predominantly physiologically- or psychologically-oriented treatment modules based on pre-treatment physiological vulnerability profiling) is needed across both categories.

#### 3.1.3 Clinical and epidemiological studies

Prospective sex-stratified ambulatory autonomic monitoring in FDS cohorts could characterise pre-ictal sympathetic trajectory and test the circulatory defence failure prediction. Systematic physiological workup of FDS patients for identifiable oxygen delivery deficits (spirometry, haematology, sleep study, cardiovascular assessment) could characterise the prevalence of potential modifiable physiological vulnerabilities. Studies with long-term follow-up periods examining the relationship between physiological vulnerability baseline profiles and responses to physiological versus psychological interventions would also be useful.

## 4 Discussion

### 4.1 Clinical implications

The following implications are conditional upon confirmation of the neuroenergetic hypothesis tested via the discriminating predictions outlined above. They are presented here as the clinical consequences of the hypothesis if confirmed, not as established recommendations.

Treatment would be fundamentally reframed. Rather than a single psychological intervention applied to a heterogeneous population, FDS management would be stratified by dominant vulnerability profile. Patients with identified physiological vulnerabilities, such as anaemia, OSA, cardiovascular disease, obesity-related respiratory limitation, or documented hyperventilation, could receive targeted physiological intervention as first line management of FDS in conjunction with or prior to psychological treatment. This is a specification and identification of which patients are most likely to benefit from specific interventions or intervention modalities, not a rejection of psychological treatment.

The diagnosis of FDS carries stigma. Some of it comes from clinical perceptions of episodes as voluntary, fabricated, or purely psychological. Some of it, patients carry themselves. A mechanistic account in which episodes are a physiological rescue response to oxygen insufficiency challenges that perspective. It also changes what the patient is being asked to do. Not to excavate a personal past or challenge a character flaw, but to address a biological component that interacts with psychology and social context to complete the biopsychosocial picture. The former has historically been disempowering and associated with treatment disengagement (75). The treatment landscape would expand to include well-established metabolic and lifestyle intervention, including dietary modification, structured exercise protocols, sleep treatment, iron supplementation, and breathing retraining, which are inexpensive, accessible, and low-risk. If these types of interventions reduce FDS frequency through the proposed mechanism, this would represent a significant expansion of the treatment toolkit for a condition which has limited intervention options.

Most accounts of FDS begin after the first episode. This one does not have to. If the threshold is loaded incrementally by factors such as obesity, sleep apnoea, physical deconditioning, and chronic stress, then the period before the first episode may be clinically visible. That reframe has practical consequences. So does the fact that the experimental questions the hypothesis generates are not waiting on new technology - the tools to answer them are already available.

## 5 Conclusion

It has been proposed here that FDS may involve a neuroenergetic deficit syndrome. The hypothesis is that FDS episodes - at least in a subgroup of patients, or as one operative pathway among several - arise when transient cerebral oxygen insufficiency is met with the brain’s inability to proactively reorganise neural networks, leading to gradient-ordered network failure, a physiological motor rescue attempt, followed by recovery upon perfusion restoration. A patient’s background vulnerability (eroded oxygen reserve from cardiovascular, haematological, respiratory, and/or stress-related sources) is the substrate upon which episodes occur. The failure to reorganise brain networks proactively under metabolic challenge is the spark. The ictal motor symptoms are the rescue, not incidental or pathological. Psychological vulnerability is not deemed to be a necessary condition for the development of FDS, but is treated as a threshold modulator under the current account. What distinguishes a hypothesis from a post-hoc narrative is whether it predicts or merely accommodates. The female preponderance is predicted here from reproductive physiology before the demographic pattern is invoked. The better prognosis in children is predicted to reflect biology that has not yet been eroded by chronic FDS episodes. The CODES result is a mechanism-treatment mismatch rather than a CBT failure. The remaining predictions are specific, falsifiable, and have not yet been tested. For patients who are told their seizures have no physical basis and are currently without an adequate mechanistic explanation for their condition, this hypothesis may be the beginning of an answer or a wrong turn. Either is progress.

## Data Availability

The original contributions presented in the study are included in the article/supplementary material, further inquiries can be directed to the corresponding author/s.
